# Pulmonary tuberculosis among people living with HIV/AIDS attending care and treatment in rural northern Tanzania

**DOI:** 10.1186/1471-2458-8-341

**Published:** 2008-09-30

**Authors:** Bernard J Ngowi, Sayoki G Mfinanga, Johan N Bruun, Odd Morkve

**Affiliations:** 1 Haydom Lutheran Hospital, Mbulu District, Manyara Region, Tanzania; 2 National Institute for Medical Research, Haydom Research Station, Mbulu, Tanzania; 3 National Institute for Medical Research, Muhimbili Medical Research Centre, Dar es Salaam, Tanzania; 4 Department of Infectious Diseases, Ulleval University Hospital, Oslo, Norway; 5 Department of Internal Medicine B, University Hospital of North Norway, Tromsø, Norway; 6 Centre for International Health University of Bergen, Bergen, Norway; 7 Department of Thoracic Diseases, Haukeland University Hospital, Bergen, Norway

## Abstract

**Background:**

Tuberculosis is the commonest opportunistic infection and the number one cause of death in HIV/AIDS patients in developing countries. To address the extent of the tuberculosis HIV coinfection in rural Tanzania we conducted a cross sectional study including HIV/AIDS patients attending care and treatment clinic from September 2006 to March 2007.

**Methods:**

Sputum samples were collected for microscopy, culture and drug susceptibility testing. Chest X-ray was done for those patients who consented. Blood samples were collected for CD4+ T cells count.

**Results:**

The prevalence of tuberculosis was 20/233 (8.5%). Twenty (8.5%) sputum samples were culture positive. Eight of the culture positive samples (40%) were smear positive. Fifteen (75%) of these patients neither had clinical symptoms nor chest X-ray findings suggestive of tuberculosis. Nineteen isolates (95%) were susceptible to rifampicin, isoniazid, streptomycin and ethambutol (the first line tuberculosis drugs). One isolate (5%) from HIV/tuberculosis coinfected patients was resistant to isoniazid. No cases of multi- drug resistant tuberculosis were identified.

**Conclusion:**

We found high prevalence of tuberculosis disease in this setting. Chest radiograph suggestive of tuberculosis and clinical symptoms of fever and cough were uncommon findings in HIV/tuberculosis coinfected patients. Tuberculosis can occur at any stage of CD4+T cells depletion.

## Background

Tuberculosis (TB) is the commonest opportunistic infection and the number one cause of death in HIV patients in developing countries, and accounts for about 40% of all manifestations seen in HIV patients [1]. About 25% to 65% of patients with HIV/AIDS have tuberculosis of any organ and tuberculosis accounts for about 13% of all HIV related deaths world wide [2-6]. While tuberculosis prevalence has declined by more than 20% worldwide, the rates in Africa have tripled since 1990 in countries with high HIV prevalence and are still rising across the continent at 3–4% per year [7]. Between 1998 and 1999, a 20% increase of tuberculosis cases was reported in countries severely affected by HIV/AIDS in Africa[8]. Correct diagnosis and treatment of tuberculosis help to reduce the burden of tuberculosis, provided that infectious cases are detected and treated successfully. However, there are difficulties in achieving the goal of reducing the tuberculosis burden due to a number of challenges, such as difficulties in diagnosing tuberculosis in HIV infected patients due to unusual clinical picture with increase in smear negative acid fast bacilli (AFB negative) pulmonary tuberculosis disease, and atypical findings on chest radiography [9,10]. People with HIV are increasingly infected with tuberculosis because HIV weakens their immune system[11] HIV/AIDS fuels the tuberculosis epidemics in many ways, such as promoting progression to active tuberculosis, increasing the risk of reactivation of latent tuberculosis infection, as well as increasing chance of tuberculosis infection once exposed to tubercle bacilli [3,12]. Autopsy studies have shown disseminated tuberculosis in 14–54% of HIV infected people in HIV prevalent countries, many of whom were undiagnosed prior to death [13]. The prevalence of tuberculosis in HIV patients on care and treatment in rural Tanzania is not known. We report on the prevalence of undiagnosed tuberculosis and drug susceptibility among the HIV patients attending care and treatment clinic in rural northern Tanzania.

## Methods

### Study design, setting and population

A cross sectional study was conducted at Haydom Lutheran Hospital in northern rural Tanzania from September 2006 to March 2007. Haydom Hospital is a 400-bed hospital in Mbulu District, owned by the Evangelical Lutheran Church in Tanzania. The hospital serves a population of about 250,000 from the surrounding three divisions [14]. The hospital has facilities for tuberculosis and HIV/AIDS diagnosis, treatment and monitoring

### Study subjects

Subjects were recruited from the patients attending the HIV care and treatment clinic. Eligible subjects were those aged 10 years and above and who agreed to participate in the study irrespective of their previous tuberculosis status. Patients on tuberculosis treatment were excluded from the study. Sputum was collected in a plastic leak-proof container and sent to the Central Tuberculosis Reference Laboratory (CTRL) for fluorescence microscopy, culture and drug susceptibility testing. All patients were able to provide sputum samples, but for those who could not produce sputum spontaneously (43 patients); we used induced sputum by inhalation of nebulised hypertonic saline. The CTRL undergo proficiency testing and external quality assessment by sending strains to a supranational laboratory in Belgium for comparison of sensitivity testing results. The time from sputum collection to the time received at CTRL was 4 days. Blood was collected in ethylene diamine-tetra-acetic acid (EDTA) tubes. The same sample was used for haemoglobin measurement and for CD4+T cells count. Tuberculosis and HIV/AIDS diagnosis, treatment and monitoring were given free of charge according to Tanzanian national policy for tuberculosis and HIV/AIDS management.

### Laboratory procedure for tuberculosis diagnosis and Haematology

At the CTRL the sputum specimens were decontaminated by using 2% sodium hydroxide (NaOH) and then concentrated by centrifugation at 3000 g. After centrifugation the samples were examined by fluorescence microscopy and also cultured on Lowenstein Jensen (LJ) medium and incubated for 8 weeks. The smear microscopy, culture and resistance test were done and interpreted according to the National algorithm for mycobacterium smear microscopy and culture[11]. CD4+T cells count were done using the Becton Dickinson Fluorescent Activated Cell Sorter (BD FACS Count, USA) haemoglobin measurement were done using Sysmex Kx-21 (Sysmex Corporation; Kobe Japan). Patients were interviewed, using a structured questionnaire. Physical examination, including anthropometric measurements (height and weight), were recorded. Chest radiography was done for all study subjects.

### Ethical consideration

The protocol was approved by the Medical Research Coordinating Committee of the Ministry of Health and Social Welfare, Tanzania. Oral informed consent was obtained from the patients prior to enrolment. For those below the age of 18 years permission was sought from parents or caretakers.

### Data management and Statistical analysis

Completed questionnaires were coded by numbers and double entered in a computer software Epi-data version 13.1. Cross-checking and data cleaning was done. The data was then transferred to SPSS version 13 for analysis[15] Categorical data were analysed by Chi square test. The level of significance was set at p ≤ 0.05, and 95% confidence interval was used throughout.

### Definition of cases

Pulmonary tuberculosis was defined as cases positive for acid fast bacilli by smear microscopy and/or culture. Anaemia was classified as haemoglobin level < 10 g/dl [16]. Immunological status was assessed by using CD4+ T cells count, and patients with CD4 + T cells < 500 cells/mm^3 ^were classified as having immunosupression[17]. Nutritional status was assessed using body mass index (BMI). Normal nutritional status was defined as BMI of 18.5–24.5 Kg/m^2 ^and malnutrition as BMI < 18.4 Kg/m^2 ^[18].

## Results

### Baseline characteristics of the study subjects

A total of 233 HIV/AIDS patients were included in the study. The mean age of the study subjects were 37, SD 10.2. Twenty (8.6%) of all patients enrolled had tuberculosis, and thirty six (15.4%) of them had a history of tuberculosis in the past 5 years. One (5%) of the tuberculosis/HIV coinfected patients and thirty five (16.4%) of the HIV positive/tuberculosis negative patients had a history of previous tuberculosis within the past five years. These patients with previous history of tuberculosis were diagnosed to have HIV when they were attending the tuberculosis clinic. One hundred and fifty (64.4%) were on ARV while 83 (35.6%) were on regular follow up. Patients on regular follow up are those who are not yet eligible for antiretroviral drugs[19]. These patients usually attend the clinic every four to six month for monitoring the progression of the HIV infection.

Baseline characteristics of the study subjects are given in table 1.

**Table 1:** Baseline characteristics of the study participants

	**Tuberculosis status**			
	
**Characteristics**	**Tuberculosis positive, N = 20**		**Tuberculosis negative, N = 213**	
	N	%	n	%
**Age**				
12–24	2	10.0	14	6.6
25–34	9	45.0	75	35.5
35–44	6	30.0	79	37.1
45–54	2	10.0	31	14.7
> 55	1	5.0	14	6.6
Mean age (SD)	35.5 (9.5)		37.4 (10.4)	
**Sex**				
Male	6	30.0	52	24.4
Female	14	70.0	161	75.6
**Marital status**				
Single	4	20.0	52	24.4
Married	10	50.0	102	47.9
Others*	6	30.0	59	27.7
**Education**				
No education***	6	30.0	81	38.0
Educated****	14	70.0	132	62.0

* Includes separated, divorced, widowed and cohabiting.

*** Includes those did not go to school at all and those who dropped before finishing 7 years of primary education.

**** Those with primary education and above, there were only two who had education above primary school.

### Chest radiograph, smear microscopy and culture

**Figure 1:**
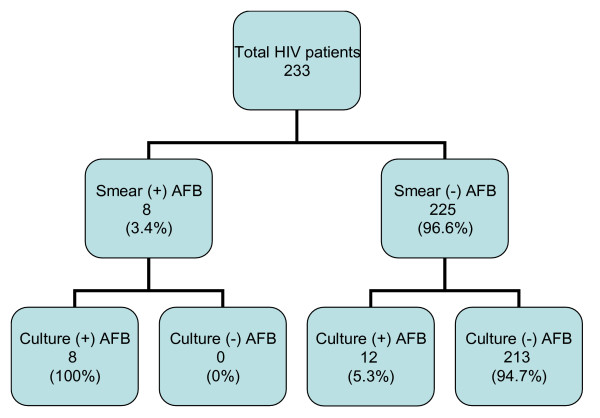
Smear microscopy and culture results for the study subjects.

Smear microscopy and culture results for the study subjects are given in figure 1.

Five (25%) of the HIV tuberculosis coinfected patients had features suggestive of tuberculosis on chest radiography; these 5 patients were also sputum smear microscopy and culture positive for AFB.

### Clinical symptoms of tuberculosis

Among patients with a diagnosis of tuberculosis by smear microscopy

5 (62.5%) had fever for > 2 weeks, 2 (25%) had cough for > 2 weeks and 4 (50%) were malnourished. All patients with smear negative AFB microscopy and culture positive AFB had no symptoms or chest radiography changes suggestive of tuberculosis.

### Smear microscopy and CD4+T cells count

One (12.5%) smear positive AFB patient had CD4+T cells of < 200/mm^3^, 3 (37.5%) had a CD4+T cells of 200–349/mm^3 ^and 4 (50%) had CD4+T cells of > 350/mm^3^.

### HIV tuberculosis coinfection and immunological status

Two thirds (3/20 (65%) of the HIV tuberculosis patients had a CD4+T cells count of < 349 cells/mm^3^. Patients who were HIV positive tuberculosis negative had higher CD4+ T cells count than the HIV tuberculosis coinfected patients. The difference between mean CD4+ T cells among people living with HIV/AIDS (PLWHA) infected with tuberculosis and those PLWHA not infected with tuberculosis is not statistically significant (p = 0.2). The CD4+T cells for the study subjects are given in table 2

**Table 2:** Immunological status of the study subjects

	**Tuberculosis status**					
	
**CD4+T Cells count**	**Tuberculosis positive N = 20**			**Tuberculosis negative N = 213**		
	n	%	95% CI	n	%	95% CI
< 200 Cells/mm^3^	6	30	9.9–50.1	72	33.8	27.5–40.2
200–349 Cells/mm^3^	7	35	14.1–55.9	48	22.5	16.9–28.1
350–500 Cells/mm^3^	4	20	2.5–37.5	36	16.9	11.9–21.9
> 500 Cells/mm^3^	3	15	-6.5–30.7	57	26.8	20.9–32.8
Mean CD4+T cells (SD)	277.9 (199)		181–368	355.0 (260)		320–390

### Drug susceptibility testing

Drug susceptibility testing was done for the 20 culture positive isolates. Nineteen (95%) were sensitive to rifampicin, isoniazid, streptomycin and ethambutol. One isolate (5%) was resistant to isoniazid, representing a patient with a history of past tuberculosis treatment, where isoniazid was one of the drugs used.

### Antiretroviral treatment and tuberculosis

Ten (6.7%) HIV tuberculosis coinfected patients were on ARV and 140 (93.3%) HIV infected tuberculosis negative patients were on ARV. Thirty three (22%) of those patients on ARV had a history of previous tuberculosis in the past 5 years, and 117 (78.0%) of the patients on ARV had no history of previous tuberculosis. The mean duration for ARV use in HIV tuberculosis coinfected patients was 6.8 months S D 9.7 and for HIV infected tuberculosis negative 9.7 months S D 10.9.

### Haemoglobin level

Five (15%) of the HIV tuberculosis coinfected patients were anaemic and 15 (75%) had normal haemoglobin level, while 52 (24.4%) of HIV infected tuberculosis negative were anaemic and 161 (75.6%) had normal haemoglobin level.

### Nutritional status assessment

Significantly more HIV tuberculosis coinfected patients were malnourished (14/20, 70%, X^2 ^= 7.2, p = 0.007) as compared with HIV positive tuberculosis negative patients 83/213 (39%). Six (30%) of the HIV/tuberculosis coinfected patients had normal nutritional status and 130 (61%) of the HIV positive/tuberculosis negative patients had normal nutritional status.

### WHO Clinical staging of HIV/AIDS disease

WHO clinical stage for the study subjects is given in table 3

**Table 3:** WHO clinical stage for HIV/AIDS for the study participants

	**Tuberculosis status**					
	
**WHO clinical stage**	**Tuberculosis positive N = 20**			**Tuberculosis negative N = 213**		
	n	%	95% CI	n	%	95% CI
I	0	0	__	23	10.8	6.6–14.9
II	0	0	__	18	8.5	4.8–12.3
III	6	30.0	9.9–50.1	70	32.9	26.6–39.2
IV	14	70.0	9.5–90.1	102	47.9	41.2–54.6

Using WHO clinical staging of HIV/AIDS disease we observed 6 (30%) of HIV tuberculosis coinfected patients in WHO stage III and 14 (70 %,) in stage IV.

## Discussion

We found high prevalence of tuberculosis disease among HIV/AIDS patients attending care and treatment in this setting. A study done in Dar es Salaam found a prevalence of 15% in patients attending HIV care and treatment clinic[20]. Our prevalence of 8.5% is low when compared with the study done in Dar es Salaam. The difference between these two studies can be explained by the fact that Dar es Salaam is urban and contributes about 24% of all tuberculosis cases in Tanzania, while the rural area where we did our study contributes less than 2%[11]. Patients with low immunity due to HIV are more likely to acquire tuberculosis in an area with high tuberculosis prevalence. Also, the prevalence of HIV in Dar es Salaam is higher than the national average of 7%[21,22] and higher than the prevalence of 2%[23] for the area where we did our study. This means that there are relatively more HIV patients who are susceptible to tuberculosis in Dar es Salaam than in the area of our study. The prevalence of 8.5% is lower than 40–54% found in autopsy studies done in HIV infected people who were undiagnosed prior to death[13] However, these studies included all forms of tuberculosis, while in our study we included pulmonary tuberculosis only. In another study done in Fajara Research clinic in Gambia among HIV patients who were followed at the clinic, 43.2% were diagnosed to have tuberculosis after 28 days of follow up, and 66% of them had pulmonary tuberculosis confirmed by microscopy and/or culture[24]. In yet another study done in South Africa among African gold miners the point prevalence of undiagnosed tuberculosis among HIV positive participants was 3.8% [25]. Our prevalence of 8.5% is close to 9% found in a study done in Cambodia [26]. Part of the differences between the prevalence in our study and the other studies may be due to differences in inclusion and exclusion criteria. As patients already diagnosed to have tuberculosis and started tuberculosis treatments were excluded in our study, this may have given lower rates than in some of the other studies.

Out of 20 tuberculosis patients in our study, smear was positive in 8 (40%) patients, this is close to the figure of 42% reported in Ethiopia [27]. Most of the mycobacteria isolated from these patients were susceptible to the first line tuberculosis drugs (rifampicin, streptomycin, isoniazid and ethambutol). Resistance to isoniazid was found in only one patient (5%), and no cases of multi-drug resistant tuberculosis was identified; this is similar to the report from the Tanzanian National tuberculosis control program, where 90% of all isolates were sensitive to these drugs and resistance to isoniazid was 5% [11]. However, another study done in northern Tanzania showed that among HIV tuberculosis coinfected patients resistance to at least one drug was 10.8% [28]. A study done in Mwanza Tanzania and published 10 years ago demonstrated that among HIV tuberculosis coinfected patients 13% had strains resistant to isoniazid, rifampicin, thiacetazone and/or streptomycin[29] A recent study from Cambodia shows that the resistance to anti tuberculosis drugs is decreasing since the introduction of antiretroviral drugs and there was a decrease of resistance from 48% in 1999 to 7.9% in 2004 [30].

Symptoms like fever, cough and weight loss were uncommon among tuberculosis patients in this study. This unusual presentation means that the screening of tuberculosis among HIV patients by using symptoms of fever, cough, weight loss and chest radiography with features suggestive of tuberculosis would detect only 25% of the HIV tuberculosis infected patients. This suggests that chest radiography and clinical symptoms, as recommended in Tanzania for follow up screening of HIV patients for tuberculosis, may not detect up to 75% of tuberculosis cases in people living with HIV. Eight patients with tuberculosis were on antiretroviral drugs for more than six months and yet developed tuberculosis; this shows that there is a need to do regular tuberculosis screening by sputum microscopy and culture to diagnose tuberculosis in HIV patients on treatment. Ten patients found to have tuberculosis were not yet eligible for antiretroviral drugs. If tuberculosis were diagnosed in these patients and correctly staged, they would be eligible for antiretroviral drugs and cotrimoxazole prophylaxis (CP) [11,19,31,32].

Anaemia was uncommon among the study subjects, 75% of them had normal haemoglobin level. This could be due to the regular check up of haemoglobin every time the patients come to the care and treatment clinic, where those who are found anaemic are treated free of charge.

Malnutrition was found in 70% of the coinfected patients. Malnutrition and tuberculosis increases morbidity and mortality in HIV patients [31,32]. Most of the HIV patients, irrespective of their tuberculosis status, were in WHO stage III and IV. This means that they present at hospital in late stage with advanced disease. Overall, females were more affected with HIV (71.1%) than males, may be due to the fact that females are more vulnerable to HIV than males because of their biological make up, and social and cultural factors.

A strength of our study is that the data come from a clinic that takes care of the HIV/AIDS patients daily, so our results reflect the real situation in a rural HIV clinic, though we have a small sample size. A limitation of our study is that it is hospital based and the patients included were those who were seriously ill. Thus, we might have missed the group of not seriously ill patients who opted not to attend the hospital for care. Also the culture media Lowenstein Jensen we used lacks sensitivity; in this case we might have missed some cases of tuberculosis. However in a resource poor setting countries like Tanzania the only available culture media is Lowenstein-Jensen media, therefore we used the available media to reflect the actual situation in this poor setting

## Conclusion

We found high prevalence of tuberculosis in this setting. Chest radiographs suggestive of tuberculosis and clinical symptoms like fever and cough were uncommon findings in HIV tuberculosis coinfected patients. Tuberculosis can occur at any stage of CD4+T cells depletion.

## Competing interests

The authors declare that they have no competing interests.

## Authors' contributions

All authors contributed to the paper, all authors conceived the study. BJN conducted the study. SGM, JNB and OM supervised the research. BJN and OM analysed the data. All authors helped to conceptualise ideas and interpret the findings. BJN prepared the draft, other authors helped to review and finalise the manuscript

## Pre-publication history

The pre-publication history for this paper can be accessed here:


